# Fathers’ experiences of feeding their extremely preterm infants in family-centred neonatal intensive care: a qualitative study

**DOI:** 10.1186/s13006-021-00394-0

**Published:** 2021-06-17

**Authors:** Evalotte Mӧrelius, Sofia Brogren, Sandra Andersson, Siw Alehagen

**Affiliations:** 1 School of Nursing and Midwifery, Edith Cowan University, Joondalup, WA Australia; 2 Perth Children’s Hospital, Nedlands, WA Australia; 3 Division of Nursing Sciences and Reproductive Health, Department of Health, Medicine and Caring Sciences, Linköping University, Linköping, Sweden; 4 Department of Paediatrics, Örnsköldsviks sjukhus, Örnsköldsvik, Sweden

**Keywords:** Breastfeeding, Fathers, Infant, Newborn, Lactation, Parents, Breast milk expression, Intensive care, Neonatal, Premature, Preterm, Sweden

## Abstract

**Background:**

Extremely preterm infants need advanced intensive care for survival and are usually not discharged before they reach the time of expected birth. In a family-centred neonatal intensive care unit both parents are involved at all levels of care including the feeding process. However, studies focusing on fathers in this situation are scarce. The purpose of this study was to explore the experiences of feeding extremely preterm infants in a neonatal intensive care unit from fathers’ perspectives.

**Methods:**

The study adopts a qualitative inductive method, reported according to the COREQ checklist. Seven fathers of extremely preterm infants (gestational age 24–27 weeks) in neonatal intensive care in Sweden were interviewed by telephone after discharge in 2013–2014. The interviews were analysed using a qualitative content analysis and confirmed by triangulation in 2021.

**Results:**

Six sub-categories and two generic categories formed the main category: “a team striving towards the same goal”. The fathers were equally involved and engaged members of the feeding team all hours of the day. The fathers shared responsibility and practical duties with the mothers, and they provided as much support to the mothers as they could. However, the fathers found it difficult to support and encourage the mothers to breastfeed and express breastmilk when the breastmilk production was low. The fathers experienced a loss when breastfeeding was not successful.

**Conclusions:**

The findings indicate that fathers want to be involved with infant care, including night-time feeds, and long and demanding feeding processes. Fathers and staff need to collaborate to provide the best support to mothers during the feeding process. This study may inspire hospital staff to acknowledge and support fathers to become more involved in the oral feeding process when an infant is born extremely preterm.

**Supplementary Information:**

The online version contains supplementary material available at 10.1186/s13006-021-00394-0.

## Background

Breastfeeding is usually seen as a female issue and most qualitative studies around breastfeeding and oral feeding of preterm infants focus on women [1]. However, when the baby is born preterm and the family is admitted to a family-centred neonatal intensive care unit (NICU), feeding the newborn infant also becomes an issue for the father [2]. If the infant is born extremely preterm, the time from birth to full oral feeding is long and complex [3]. Like mothers, fathers are expected to take part in the feeding all hours of the day. Currently, there are few published studies on fathers’ experiences of oral feeding in the neonatal intensive care unit.

The European Association for Children in Hospital (EACH) emphasises that all children have the right to have their parents (or parent substitute) with them at all times while in hospital [4]. The EACH charter relates to the UN Convention on the Rights of the Child in which article seven stipulates that children have the right to be cared for by his/her parents from birth [5] and to receive family-centred care (FCC) [6]. Family-centred care is an approach in health care based on a mutual collaboration between child, family and staff [7]. In FCC, the care is focused on the family as a unit [8]. Family-centred care is an important approach to ensure that the EACH charter is followed when a child is hospitalised. However, even though FCC is implemented and accepted in paediatric hospitals all over the world, it differs when it comes to neonatal intensive care [9, 10]. For instance, a survey from Spain in 2018, found that only 21.5% of the 65 NICUs included in the survey practiced free parental access [11].

The World Health Organization estimates that 15 million infants are born premature (< 37 weeks of gestation) every year [12]. The one-year survival rate among extremely preterm infants born before 27 weeks of gestation is more than 75% and constantly improving [13]. However, between 2004 and 2013 in Sweden, the prevalence of exclusive breastfeeding among extremely preterm infants at discharge from the NICU, declined from 55 to 16% [14]. Mothers are keen to provide their breastmilk to their extremely preterm infants but the journey from birth to exclusive breastmilk feeding is long and filled with struggle and hindering factors such as stress and uncertainty about the outcomes for the infant [3, 15]. Mothers have stated that they need support when practicing breastfeeding and milk expression, and that they often get better support from their partners than from hospital staff [15]. Despite the fact that fathers are involved in FCC neonatal care [2, 16], little is known about their experiences of oral feeding extremely preterm infants in the NICU [3].

The role of the father of an extremely preterm infant in the NICU may be influenced by the context, country of origin and personal preferences [17]. A recent study involving fathers of full-term infants from Singapore, showed that fathers’ involvement in breastfeeding was influenced by perceived approval of family members and friends, fathers’ knowledge of breastfeeding and marital relationships [18]. However, becoming a father to an extremely preterm infant hospitalised in a family-centred NICU for months provides a different perspective and context as the fathers are more involved in the care. Feeley et al. (2013) suggest three kinds of involvement among fathers in the NICU: fathers may see themselves as equal to the mother; find the mother to be more important than themselves; or are reluctant to be involved in care [19].

A systematic review of lived experiences of fathers in the NICU comprising 14 qualitative studies found that the fathers experienced an “emotional roller-coaster” in the NICU, and identified paternal needs as including being informed, treated with respect and being recognised as fathers [20]. They used different coping strategies such as hiding their own feelings or hiding at work. Fathers who engaged in care-giving activities, such as skin-to-skin contact, were more likely to be aware of their role as a father and to have an easier transition into parenthood [20]. Even though some studies have focused on fathers’ experiences of neonatal intensive care [20], few studies have exclusively included fathers of extremely preterm infants [21]. Moreover, to our knowledge no previous study has focused on the oral feeding process as a care-giving activity for fathers of extremely preterm infants in the NICU.

### Aim

The aim of this study was to explore the experiences of feeding extremely preterm infants in a neonatal intensive care unit from the fathers’ perspectives.

## Methods

### Design

This explorative study used a qualitative method with an inductive approach [22].

### Setting

This study was conducted in Sweden where approximately 500 infants are born extremely preterm each year [13]. There are six hospital regions with NICUs providing equal care for extremely preterm infants according to national guidelines [23]. Both parents are expected to stay at the hospital and care for their infant as primary caregivers. However, nurses are responsible for the advanced care of the infant [24] as well as providing breastfeeding and lactation support. Parents are entitled to 480 days of paid parental leave in Sweden. To increase the opportunity of parents staying with their premature infant in the NICU all hours of the day, parents are also entitled to a temporary parental allowance when a child is sick or born preterm.

### Inclusion criteria

The inclusion criteria involved Swedish speaking fathers of extremely preterm infants born without major malformations that could potentially affect feeding. At the time of the interview, more than a month discharge was required to ensure that the family had settled at home.

### Procedure

Following ethical approval, heads of departments and nurse managers from the six regional NICUs in Sweden caring for extremely preterm infants were contacted. Five NICUs agreed to participate and appointed a contact nurse at each site. The contact nurses identified potential participants and invited their participation. Twelve fathers received written and oral information about the study; eight gave informed consent to participate and be contacted by the interviewer. It was not possible to contact one father at the time of the interviews. However, because of the narrow study aim, a specific target group, and the rich data generated from the seven interviews, data saturation was reached and the inclusion was closed [25].

### Data collection

Data were collected through telephone interviews recorded by one of the authors. Each interview started with an overview of the purpose of the study and an informal chat. The interview was guided by open-ended questions (see Additional file 1). The first question asked fathers to talk about their experiences of feeding their extremely preterm infants in the NICU before progressing to questions about the participants’ involvement, role and support, employing prompts such as, “can you elaborate on that?” and “how did you feel about that?” to enrich their stories. The interviews were conducted between September 2013 and March 2014 and ranged between 15 to 60 min with an average duration of 30 min.

### Data analysis

The interviews were transcribed verbatim by one of the authors and analysed in phases according to an inductive content analysis [22] by another author. In the first phase, the interviews were read several times to make sense of data and create an overview of the content. Meaning units in the form of statements and phrases were then extracted from the transcripts. In the next phase extracted meaning units were coded and condensed. In the following phase the codes were abstracted and, based on similarity, sorted into sub-categories, generic categories and finally into a main category. The analysis involved a back-and-forth process between authors to reach a final agreement. An example of the data analysis is described in Table 1.

**Table 1:** Example of data coding and analysis process

Meaning units	Condensed code	Sub-category	Generic category
“I believe we hadn’t thought much about that, but we thought she should have breastmilk, at least.”	Obvious to give breastmilk	Be prepared	Shared responsibility for the feeding process
“We breastfed first and then we tube-fed /…/because he couldn’t suckle so much. I prepared all the breastmilk; heated that; and changed the nappy and put him to the breast. He had a lot of cords, oxygen, and things. It was paper journals too, to document everything, I took care of that too.”	Helped each other	Working together	
“Actually, the biggest problem was when we decided that we should quit pumping, so to say…”	Decided together to stop milk expression	Share decisions	
“[It is] Pretty usual that the milk production decreases after a couple of weeks, it happened both times for [wife’s name]. It’s kind of difficult to ask her to fight then. Because it interrupts the night pretty much, she must get up every third hour to pump and then find there is no output.”	Difficult to ask her to fight when there was no breastmilk	Supporting the partner	A long and demanding process
“The only advantage with the feeding tube was that we could feed her while she was sleeping. So that was an advantage during the night. We could just wake up by the clock, feed her, and go back to bed and she slept the whole time.”	Gavage feeding meant more sleep	Full-time work	
“At the moment, yesterday and today, we try, for an intensive period, to breastfeed again.”	Tried breastfeeding again when home	A long process	

### Trustworthiness

The transparency of the content analysis and the inclusion of all authors in the analysis process helped to establish trustworthiness. Quotations were used to illustrate examples of the results to give the reader an opportunity to evaluate the concordance between the interviews and the categories identified. Three of the authors were paediatric nurses, of which two have clinical experience of neonatal intensive care and the other was a midwife with experience in care of new families but not neonatal intensive care. All authors are female.

To ensure the trustworthiness of the findings, a data source triangulation [26] was performed. Contact was made with the President of the Swedish Society for Prematurity (Svenska prematurförbundet). This contact resulted in two fathers volunteering to read the findings and share their reflections. Both fathers had extremely preterm infants born in January 2019, and experiences from different NICUs. Their experiences were very similar to those of the fathers’ interviewed in this study. The volunteer fathers recognized experiences across the categories, thereby confirming the current study findings and indicating that the findings of the 2013–2014 study retain its clinical importance.

### Ethical considerations

The study was approved by the Regional Ethical Review Board (Dnr 2012/162–31) as following the Declaration of Helsinki [27]. The results are reported in accordance with the Consolidated Criteria for Reporting Qualitative Research (COREQ) checklist (see Additional file 2) [28].

## Results

### Participants

The fathers mean age was 32 years, with a range of 27–37 years and resided with their infant’s mother. Four babies were boys and three were girls born between 24 and 27 weeks gestation. Two of the fathers had older children, one of which was born preterm.

### A team striving towards the same goal

The main category “a team striving towards the same goal”, comprised two generic categories and six sub-categories (see Table 2) identified the father as an important part of the feeding team of the mother and extremely preterm infant. The fathers were involved, engaged and wanted to be part of the NICU care, including the feeding process. The fathers cherished and valued this experience as it provided opportunities to learn about and bond with their newborn infant. They shared equal responsibility with the mothers and supported the mother and infant as much as possible. However, they experienced difficulties when attempting to support the mothers while breastfeeding and expressing breastmilk as they found it difficult to push the mother when observed her struggling to breastfeed day and night with a decreasing milk supply. The fathers worked hard around the clock to support the feeding process and experienced a sense of loss when the breastfeeding was not successful.

**Table 2:** Summary of sub-categories, generic categories and main category

Sub-categories	Generic categories	Main category
Be prepared	Shared responsibility for the feeding process	A team striving towards the same goal
Working together		
Share decisions		
Supporting the partner	A long and demanding process	
Full-time work		
A long process		

### Shared responsibility for the feeding process

#### Be prepared

The fathers had not discussed breastfeeding with their partners before the birth of their preterm baby, either because there was no time because of the interrupted pregnancy or it was not something to discuss because it was natural and obvious that the infant should be breastfed. Fathers indicated that breastfeeding was their preferred choice for feeding because it was easy and convenient and was good for the baby.

*We didn’t have time to prepare ourselves, or talk about so much, or think it should be like this (Father A)*

Fathers were more prepared if they had children who had been breastfed. These fathers reported that it was natural that the baby should be breastfed and was not really something to talk about. However, even if they expected their baby to be breastfed from the beginning, they understood that it was more important that their infant received breastmilk than being fed from the breast.

#### Working together

The feeding process was shared between the fathers and mothers. When the fathers talked about their preterm infants’ feeding and shared responsibilities with the mother, they talked in terms of “us” and “we”. They showed interest and engaged in the feeding process together with the mother. The fathers spoke about how, as a couple, “they” had practiced breastfeeding, tried different ways of feeding, inserted the nasogastric tube, and expressed milk. They were so involved in the feeding that it was almost as if they had breastfed and expressed breastmilk themselves.

*We started with the breastfeeding already in the intensive care room (Father D)*

The fathers’ involvement in their infants’ feeding process was obvious when they talked about their experiences and awareness of the struggle feeding an extremely preterm infant.

*We wish we had managed to get breastfeeding to work earlier (Father B)*

The fathers participated in the feeding process just as much as the mothers but in slightly different ways. As they could not breastfeed or express breastmilk, they administered more gavage feeds and changed the nappies. On the ward, parents were allowed to carry out most of the care for their infant including every step in the feeding process. The fathers wanted to be involved in their infants’ care and described the feeding process as a perfect task as it provided a positive opportunity to get to know and bond with their infant. On occasions an infant needed food every second hour around the clock. To handle that situation, the parents shared the feeding. Although gavage feeding was easy to share, the fathers emphasised the importance of continuing to share feeding responsibilities once the infant no longer needed gavage feeding. When the mother was practicing breastfeeding with her infant, both parents were in attendance day and night to help and assist each other.

*. . . we did it [the feeding procedure] together all the time, [because] it was so inconvenient to do it by yourself . . . you had to tube feed and check the position of the tube and all, so it was inconvenient to do everything by yourself. So, we did it together instead (Father C)*

Their involvement helped fathers’ confidence when talking about food, breastfeeding, and milk expression. Fathers showed an awareness of different types of formula; how much milk the infant was given at each meal; and how the intake of milk increased each day, based on the infant’s weight. This awareness could only be obtained through experience.

*She sometimes takes it [the pacifier] very well and it doesn’t seem to disturb her way of, how to say, how good she takes the breast (Father B)*

If there were siblings, the shared responsibility evolved a bit differently if one parent needed to pay more attention to the sibling than to the preterm infant.

*He [the brother] stayed with us at the hospital so it was natural that we shared all tasks, one of us was with the girl and one with the boy (Father E)*

If the sibling had also been born preterm and admitted to NICU, it was more difficult for the father to recall details about each infant’s feeding, for instance for how long the mother continued to express breastmilk.

#### Shared decisions

Both parents mostly agreed on decisions about their infant’s feeding. The fathers’ involvement was evident through the shared responsibility of decision making. To stop expressing milk was one of the hardest decisions for the fathers to make. There was a sense of disappointment when they talked about this decision even if it was a shared one and felt correct at the time as fathers wondered what might have been if they had tried a bit longer; maybe the infant would eventually have managed to suckle?

*Eventually, when she had pumped for weeks without any milk discharge, it was no fun to continue, it only hurts for nothing, so to say. So she stopped pumping (Father F)*

Most of the time, changes around feeding were shared decisions. But in some cases, fathers took on a support role and reinforced the decision of the mother.

*In some way I allowed my wife to express her views first and then I supported her decision. I felt she was very grateful. Most of the time I let her decide, and I agreed because they were wise decisions she made (Father E)*

Some parents never took a final decision but rather kept on going and took each day as it came, 1 day at a time. If no decisions were made, the hope was still there.

*Well, we never really stopped. We tried with the breastfeeding for a while, even though we knew it didn’t work out very well. It was because he should have [breastmilk], we thought he might learn one day . . . I can’t remember for how long we kept going . . . (Father D)*

### A long and demanding process

#### Supporting the partner

Being supportive as a father was sometimes a difficult task. It was important to make sure the mother was both physically and emotionally well. Some mothers needed physical support after they had given birth, especially if they had a caesarean section, had lost a lot of blood, or had other complications. The mothers started expressing milk early on and continued to do this regularly. The father was available to undertake extra feeds to allow the mother time to eat, have some time out or to let her rest or go home.

*Sometimes I took care of an extra feed if she [mother’s name] needed to plan the day according to the pumping [schedule] (Father A)*

Emotional support could also involve encouraging a mother with previous experience of losing a preterm infant to interact with her newborn infant. Emotional support also involved showing a respectful approach, and letting the mother take the first step towards feeding the infant.

*From the beginning everything happened automatically but later on when we could start to feed [the baby] by ourselves, then I let my wife take the first step in trying to feed the baby (Father E)*

There was always something happening around the infant and the fathers described how the mothers sometimes seemed stressed because they reacted in a sensitive way to staff comments or the tone of the staff voices.

Being a father during breastfeeding practice and milk expression was especially difficult as they could not take over or help. They tried to be present and to provide a helping hand if needed, but they had trouble in finding a suitable way to support the mother during these moments.

*Yeah, I did the best I could, as I felt I could. It is difficult to do something special to help, I think. Really, there is not so many ways of helping. I did the best I could (Father D)*

The fathers encouraged and praised the mothers when everything worked well, but if there was no milk, the fathers found it hard to reassure, encourage and support the mothers to continue to express milk and practice breastfeeding every 3 hours.

*Well, it was hard for her to pump milk and do all that, and [for me to] try to support [her] when the breastmilk ceased . . . and say it is not her fault (Father G)*

The fathers indicated that the collaboration between them and the staff was less than optimal. They tried to be supportive and protective towards the mothers when the staff increased the pressure on the mothers by repeatedly querying their progress with the expression.

#### Full-time work

The feeding process was a full-time job with each feeding session taking up to an hour. Consequently, both parents needed to be at the hospital all hours of the day to make sure milk expression, breastfeeding practice, gavage feeding, and documentation were carried out as efficiently and accurately as possible. The father’s task during the feeding procedure was to change the nappy, hand the baby over to the mother to practise breastfeeding and, in the meantime, prepare previously expressed milk for gavage feeding to top up the breastfeeding.

*. . . and at the same time, you should try to get some rest too (Father E)*

Gavage feeding was a big advantage during the night as parents could feed the infant while he/she was still asleep and quickly return to bed.

Being active around the clock affected both parents’ sleep as they were both awake during all feedings. It was difficult for them to get several hours of sleep in a row because of the frequent feeding schedule. Only one of the fathers described how he and his partner asked the staff for help with the feeding so they could get some continuous sleep.

*We asked for help. We sought [help] when we hadn’t slept more than one hour in two days, we couldn’t manage more so we asked for help. That was no problem, they were happy to help. So nice, they took care of him for two feedings and we could sleep four, five hours. Made a huge difference (Father C)*

The situation of having an extremely preterm infant was difficult for both parents who described the situation as mentally difficult, worrying, exhausting, and filled with shared tears.

#### A long process

Being a father and caring for an extremely preterm infant included many weeks of in-hospital stay, which involved sleep loss, worry, and stress. Later, when the infant was on formula and/or the infant’s health status was more stable, the fathers who had other children were able to leave the hospital to care for older siblings at home. Other fathers continued with house renovations or took some time out for a couple of days.

*Well our son [the brother] and I moved home for the last month, we lived 30 km from the hospital (Father D)*

However, the struggle with food was far from over for some of the fathers. They witnessed a continual battle when trying to encourage an extremely preterm infant to eat. Once at home with the infant parents had contact with a dietician and a child health nurse about feeding or tried to get ideas from the internet or magazines for new parents. One child was gavage fed through a gastrostomy. Another father described how he and his wife were trying to re-establish breastfeeding following discharged from the hospital, giving it one more last chance.

## Discussion

The fathers shared equal responsibility with the mother when it came to feeding the extremely preterm infant. The fathers were involved, engaged, and needed during the feeding process. Their involvement gave them confidence to talk about infant feeding, including breastfeeding and milk expression. In a Canadian study of fathers in the NICU, the fathers showed three different involvement patterns: fathers who perceived themselves as equal to the mother; found the mother to be more important than themselves; or were reluctant to be involved in the infant’s care [19]. The fathers participating in the present study saw themselves as equal to the mother regarding responsibilities around feeding. This attitude might be an effect of the FCC where both parents are invited to stay at the hospital all hours of the day, due to a healthcare system that supports time off from work. This finding is consistent with a study by Feeley and colleagues showing that paternity leave and employment leave facilitated involvement in care [17]. Moreover, in a study from Ghana where fathers do not have the possibility for FCC, the fathers experience was that the NICU was for the mothers only and they did not feel involved in the care. Consequently, they felt incompetent to feed their infants after discharge [10].

The results show that being a father in family centred NICU and sharing the responsibility for feeding with the mother is also a full-time job as it involves feeding the infant six to twelve times a day for several months. This time is also filled with worry and uncertainty about the infant’s and mother’s health and can be described as an emotional “roller-coaster” [20, 29]. The combination of being on an emotional “roller-coaster” and feeding an infant several times during the night increases the risk of sleep loss and exhaustion [30]. Consequences of sleep loss have previously been described among parents of sick children in terms of cognitive issues such as difficulty in the ability to concentrate, solving problems, and comprehending information [30, 31]. Sleep loss and exhaustion are also potential stressors that can reduce the possibility of breastfeeding [32]. Only one of the fathers in the present study mentioned that they had asked the staff to help with care for their infant in order to get some sleep. Previously, parents in a Swedish NICU have described tiredness as something you should expect as a new parent [30]. It is possible that such expectations make parents hesitant to ask for help. Potential sleep loss is a drawback of the FCC which needs to be addressed; one way could be to assist the parents though drawing up a structured plan on how they can take turns with feeding.

Mothers have previously described finding their partners to be the most important source of support while establishing breastfeeding and expressing breastmilk [10]. This finding is in line with the present study which found that fathers did everything they could to praise and support the mother. However, the fathers struggled when it came to supporting breastfeeding and breastmilk expression, especially when the breastmilk ceased. The fathers found it hard to encourage the mother to continue when they saw her struggling to produce breastmilk. Under these circumstances the fathers had no control over the situation as they were unable to step in and take over in order to help. One possible reason for feeling unable to support could be lack of knowledge about breastfeeding and how to support mothers in the best way possible [18]. A more effective approach would be for the fathers and staff to work together to find individual solutions to support the mothers with breastfeeding and breastmilk expression.

The fathers gave no sign of hiding their own feelings, as previously described as a coping strategy among fathers in the NICU [20, 33]. Instead, the fathers described how they had cried, wished for breastfeeding to work, and experienced disappointment when breastfeeding was not successful. Breastfeeding a preterm infant in the NICU is no longer a female only issue. Therefore, it is important to also acknowledge and support fathers’ feelings in the NICU and, more specifically, around breastfeeding. Moreover, the ten steps to successful breastfeeding [34] need to be updated to include the father/partner since they are an important member of the feeding team.

No claims are made to transfer the findings in this study beyond the present context. However, some limitations need to be highlighted. We do not know if the convenience sampling method has affected the findings. For instance, it is possible that the fathers declining to participate were fathers that were less involved in the feeding process than the participating fathers, which can potentially affect the findings. Another limitation is that no female partners participated. Although the data is from some years ago the findings are still of clinical importance which was confirmed by the triangulation, since fathers are getting more involved in the care of the infant when FCC is applied in the NICU.

## Conclusions

The fathers were equally engaged and involved as the mothers in the feeding process. However, they found it difficult to support the mothers with breastfeeding and breastmilk expression, when the breastmilk supply was ceasing. Fathers in family-centred NICUs need to be acknowledged. There is also the need for more emphasis on collaboration between fathers and staff to find optimal individual solutions to handle the feeding process of extremely preterm infants. This study may encourage neonatal staff to support fathers to become more involved in the NICU care of their infants and the feeding process.

## Supplementary Information

**Additional file 1.** Interview guide.

**Additional file 2.** Consolidated Criteria for Reporting Qualitative Research (COREQ) checklist.

## Data Availability

The datasets used and/or analysed during the current study are in Swedish and available from the corresponding author on reasonable request.

## Abbreviations

FCC: Family-centred Care
NICU: Neonatal Intensive Care
