# Supplementary material for: Fathers’ experiences of feeding their extremely preterm infants in family-centred neonatal intensive care: a qualitative study
Source: Int Breastfeed J. 2021 Jun 17;16:46. doi: 10.1186/s13006-021-00394-0 (PMC8212537; doi:10.1186/s13006-021-00394-0)
Supplement: Supplementary file 1 — Additional file 1. Interview guide. [file 13006_2021_394_MOESM1_ESM.docx]

Additional file 1. Interview Guide

**Topic Guide question**

| Purpose of the study | The aim of this study is to explore the experiences of feeding extremely preterm infants in a neonatal intensive care unit from fathers’ perspectives. |
| --- | --- |
| Clarifications | When we talk about the ‘feeding process’, ‘food’ and ‘feeding’ in this study, it includes milk expression, gavage, breastfeeding, breastfeeding practice, and cup/bottle-feeding. |
| Descriptive questions | Age, marital/cohabitate status, number of children in the family, previous experience of premature birth, gender of child born preterm, gestational age of child born preterm, time since discharge from the neonatal unit. |
| Main areas to discuss | Please tell me about your experiences of feeding [child’s name] in the neonatal unit.  Please tell me about your involvement in [child’s name] feeding process.  Please tell me about your role in [child’s name] feeding process.  Please tell me about your support needs around the feeding process in the neonatal unit.  Were there any positive or potential problems you experienced around the feeding process.  Please tell me about your experiences of feeding [child’s name] after discharge from the neonatal unit. |
| Closing | Is there anything else that hasn’t come up that you would like to add? |
| Probing questions | Can you elaborate on that…  How did you feel about that…  Can you tell me more about…  Can you give an example of…  How was that… |
